# The endogenous HBZ interactome in ATL leukemic cells reveals an unprecedented complexity of host interacting partners involved in RNA splicing

**DOI:** 10.3389/fimmu.2022.939863

**Published:** 2022-08-01

**Authors:** Mariam Shallak, Tiziana Alberio, Mauro Fasano, Maria Monti, Ilaria Iacobucci, Julien Ladet, Franck Mortreux, Roberto S. Accolla, Greta Forlani

**Affiliations:** ^1^ Laboratories of General Pathology and Immunology “Giovanna Tosi”, Department of Medicine and Surgery, University of Insubria, Varese, Italy; ^2^ Laboratory of Biochemistry and Functional Proteomics, Department of Science and High Technology, University of Insubria, Busto Arsizio, Italy; ^3^ Department of Chemical Sciences, University Federico II of Naples, Naples, Italy; ^4^ CEINGE Advanced Biotechnologies, Naples, Italy; ^5^ Laboratory of Biology and Modeling of the Cell, CNRS UMR 5239, INSERM U1210, University of Lyon, Lyon, France

**Keywords:** HTLV-1, HBZ, ATL, interactome, alternative splicing, protein network

## Abstract

Adult T-cell leukemia/lymphoma (ATL) is a T-cell lymphoproliferative neoplasm caused by the human T-cell leukemia virus type 1 (HTLV-1). Two viral proteins, Tax-1 and HBZ play important roles in HTLV-1 infectivity and in HTLV-1-associated pathologies by altering key pathways of cell homeostasis. However, the molecular mechanisms through which the two viral proteins, particularly HBZ, induce and/or sustain the oncogenic process are still largely elusive. Previous results suggested that HBZ interaction with nuclear factors may alter cell cycle and cell proliferation. To have a more complete picture of the HBZ interactions, we investigated in detail the endogenous HBZ interactome in leukemic cells by immunoprecipitating the HBZ-interacting complexes of ATL-2 leukemic cells, followed by tandem mass spectrometry analyses. RNA seq analysis was performed to decipher the differential gene expression and splicing modifications related to HTLV-1. Here we compared ATL-2 with MOLT-4, a non HTLV-1 derived leukemic T cell line and further compared with HBZ-induced modifications in an isogenic system composed by Jurkat T cells and stably HBZ transfected Jurkat derivatives. The endogenous HBZ interactome of ATL-2 cells identified 249 interactors covering three main clusters corresponding to protein families mainly involved in mRNA splicing, nonsense-mediated RNA decay (NMD) and JAK-STAT signaling pathway. Here we analyzed in detail the cluster involved in RNA splicing. RNAseq analysis showed that HBZ specifically altered the transcription of many genes, including crucial oncogenes, by affecting different splicing events. Consistently, the two RNA helicases, members of the RNA splicing family, DDX5 and its paralog DDX17, recently shown to be involved in alternative splicing of cellular genes after NF-κB activation by HTLV-1 Tax-1, interacted and partially co-localized with HBZ. For the first time, a complete picture of the endogenous HBZ interactome was elucidated. The wide interaction of HBZ with molecules involved in RNA splicing and the subsequent transcriptome alteration strongly suggests an unprecedented complex role of the viral oncogene in the establishment of the leukemic state.

## Introduction

The human T cell leukemia virus type-1 (HTLV-1) is the first discovered human retrovirus (1) and the etiologic agent of a severe, still uncurable T cell leukemia of the adult (ATL) (2) and of a series of chronic inflammatory diseases among which HTLV-1-associated myelopathy/tropical spastic paraparesis (HAM/TSP) is the most debilitating pathology (3, 4). HTLV-1 infects at least 10-15 million people worldwide with a typical geographic distribution in Japan, sub-Saharan area, the Caribbean, south American countries and central Australia (5). HTLV-1 infection is transmitted by cell-to-cell contact mostly by breast feeding, blood transfusion and sexual intercourse (6). ATL develops in about 5% of the infected individuals after a long period of incubation that can last decades (7). Given this high incidence of tumors, HTLV-1 is considered as the most oncogenic pathogen to date (8).

Two viral products, the Tax-1 transactivator and the HTLV-1 basic leucine zipper factor (HBZ) play an important role in the genesis and maintenance of the oncogenic process by disarranging basic mechanisms of cell activation (9, 10). For example, Tax-1 constitutively activates NF-κB pathway and suppresses the DNA repair mechanism, thus favoring the accumulation of mutations essential for the establishment of the oncogenic phenotype (11). Previous studies indicated that Tax-1 is not expressed in 40% of ATL suggesting it may act in the initiation but not in the maintenance of the oncogenic process (12, 13). However this percentage maybe an overestimate, as Kataoka and coworkers have shown that an exceedingly low number of the 57 ATL patients genetically characterized in their study have lost Tax-1 expression (14). Moreover the fact that most of those patients still maintain Tax-interacting factors together with the recent evidence from Mahgoub et al. (15) partially challenge this notion. Indeed, they found that ATL cells with no structural alteration of the *tax* gene, display discrete bursts of Tax-1 expression. Furthermore, knockdown of Tax-1 expression induced apoptosis of ATL cells, suggesting that sporadic burst in Tax-1 expression favors the growth of the ATL population (15).

On the other hand, HBZ is always expressed in all ATL suggesting it may contribute to the stabilization and persistence of the tumor state (16, 17). Although a large body of information has been obtained about crucial interactions of Tax-1 and HBZ with factors regulating cellular activation and host gene expression (18), a clear picture of the cellular and molecular mechanism at the basis of the progression from infection to the establishment of cancer is still largely incomplete. Within this frame, an important prerequisite is the clarification of the subcellular localization of Tax-1 and HBZ during infection and disease progression as this parameter can be strictly associated with change in the pattern of interaction of the viral oncogenes with host factors governing cell homeostasis. It has been found that Tax-1 is generally expressed in both cytoplasmic and nuclear compartments throughout the various stages of infection to ATL transformation. Conversely, recent studies of our group have clearly shown that HBZ is an exclusive cytoplasmic protein in recently infected still asymptomatic carriers, persists in this subcellular compartment in patients affected by HAM/TSP and importantly, progressively dislocates in the nucleus in leukemic cells of ATL patients, representing therefore an unprecedented marker of oncogenic progression (19–22). Given these premises, we have undertaken a structured analysis of the endogenous HBZ interactome in leukemic cells, starting with the HBZ nuclear interactome, to assess the extent, the specificity and the functional role of the HBZ interacting partners particularly in relation to oncogenic transformation. In this endeavor we were greatly facilitated by the use of a monoclonal antibody specific for HBZ isolated by our group and capable to detect endogenous HBZ in HTLV-1 infected cells and in ATL (19).

The results presented in this investigation demonstrate a rather complex pattern of interactions of HBZ with a large number of intracellular factors, mainly belonging to components of RNA splicing and stability, key mechanisms controlling gene expression and cell homeostasis.

Alterations of the above mechanisms by HBZ are therefore strongly suspected to play a crucial role in the process of HTLV-1-dependent oncogenesis.

## Materials and methods

### Cells

ATL-2, an established cell line derived from a leukemic patient infected by HTLV-1 (12, 23) was kindly donated by Dr. M. Matsuoka, Kumamoto University, Japan. ATL-2, MOLT-4 and Jurkat T cells were cultured in RPMI supplemented with 10% heat inactivated fetal calf serum (FCS). Jurkat-HBZ expressing cells were maintained in complete RPMI medium under puromycin selection (5µg/ml). The 293T human embryo kidney cell line was grown in Dulbecco modified Eagle medium (DMEM) supplemented with 10% FCS.

### HBZ immunoprecipitation

Nuclear and cytoplasmic fractions were prepared from ATL-2 (40x10^6^ cells), Jurkat-HBZ expressing cells (5x10^6^ cells) and Jurkat cells (5x10^6^ cells) using NE-PERTM Nuclear and Cytoplasmic Extraction Reagents (Thermo Fisher Scientific) according to the manufacturer’s instruction. Five percent of nuclear and cytoplasmic extracts were resolved by 9% sodium dodecyl sulfate-polyacrilamide gel electrophoresis (SDS-PAGE) and analyzed by Western blotting with anti-Nup98 (Cell Signaling Technology) and anti-β-tubulin (Sigma-Aldrich) mAbs to assess the purity of nuclear and cytoplasmic fraction, respectively. HBZ proteins were precipitated from both nuclear and cytoplasmic fraction using the anti-HBZ 4D4-F3 mAb. Briefly, after preclearing with protein A-Sepharose beads and protein G-Agarose beads, cell extracts were incubated with anti-HBZ 4D4-F3 mAb for 1h and immunoprecipitated with protein A-Sepharose beads and protein G-Agarose beads overnight at 4°C.

### Mass spectrometry analysis

Following anti-HBZ immunoprecipitation in ATL-2 cells, HBZ and its interactors were eluted from the beads, resolved onto a 10% SDS-PAGE and processed by a classical proteomics protocol for protein identification. From the SDS-PAGE gel stained with colloidal Coomassie, 14 slices were excised from the sample lane (immunoprecipitation) and, in parallel, from the control lane (preclearing of immunoprecipitation). Each band of sample and control was subjected to an *in situ* trypsin hydrolysis procedure (24) and the peptide mixtures were analyzed by nano-Liquid Chromatography-tandem Mass Spectrometry (nano-LC-MS/MS) using an LTQ Orbitrap XL mass spectrometer (Thermo Fisher Scientific Inc., Waltham, MA, USA) equipped with a Proxeon nanoEasy II capillary High Performance Liquid Chromatography (HPLC). The nano-LC-MS/MS analyses were performed as described elsewhere (25). The raw data were processed into mgf files and proteins identified by means of Mascot licensed software (Matrix Science Boston, USA) using the Homo Sapiens National Center of Biotechnology Information (NCBI) database. The mass tolerance was set at 10 ppm for MS and 0.6 Da for MS/MS search. Carbamidomethyl (C) was used as fixed modification and Gln->pyro-Glu (N-term Q), Oxidation (M), Pyro-carbamidomethyl (N-term C) as variable modifications.

Classification of HBZ interactors was obtained by the Gene Ontology slim (GOslim) classification on the Webgestalt platform (http://www.webgestalt.org).

### Functional enrichment analysis

All protein lists were analyzed by over-representation analysis using Reactome as the pathway database on the Webgestalt platform (other parameters: reference database, genome protein-coding; False Discovery Rate (FDR) <0.05 after Benjamini-Hochberg correction; redundancy reduction, “Affinity Propagation” algorithm (26)). Pathways were ordered by FDR and only the top 10 enriched pathways are presented.

### Network analysis

Cytoscape 3.8.2 was used to generate a protein-protein interaction (PPI) network (27). The public database Intact was queried through Cytoscape using the Proteomics Standard Initiative Common QUery InterfaCe (PSICQUIC), by searching the HBZ interactors identified by the MS analysis. The network was filtered for taxonomy ID 9606 (Homo sapiens) to remove homology inferences. All self-loops and duplicated edges were removed.

Identifiers (IDs) of the original list have been selected on the whole network and the sub-selection extracted (to visualize known interactions among HBZ identified interactors). The GLay algorithm has been used to cluster the network in subnetworks. Each of them has been analyzed by over-representation analysis by the Webgestalt platform.

### Production of viral vectors containing HBZ

Myc tagged HBZ-expressing vector (pAIP-HBZ) for lentiviral transduction, was obtained by cloning the HBZ cDNA obtained from pcDNA3.1 Myc-His HBZSP1 vector (kindly donated by Dr. Mesnard (28), into the pAIP transfer vector that expresses Myc-His HBZ as part of a bicistronic RNA driven by the Spleen Focus-Forming Virus (SFFV) Long Terminal Repeat (LTR) promoter, containing the encephalomyocarditis virus internal ribosome entry site and a puromycin resistance cassette (Adgene). The HBZ coding sequence was amplified by PCR using the following primers:

HBZpAIP_SE: 5’GCGAATTCATGGCGGCCTCA3’

HBZpAIP_AS: 5’GCGAATTCTCAATGATGATGATGATGATG3’

and cloned into EcoRI site of the PAIP vector (Adgene).

### Lentiviral vector production and generation of HBZ stably expressing Jurkat cells

Lentiviral vectors were produced by co-tranfecting 293T cells seeded at 6×10^5^ cells/ml in six-well plates with pMD2.G, psPax2, and pAIP-HBZ or pAIP empty vector at 1:3:4 ratios by using FugeneHD (Promega). Vector containing supernatants were harvested at 24h and 48h post-transfection and cleared by centrifugation. In order to generate Jurkat-HBZ expressing cells, Jurkat T cells were transduced twice with pAIP-HBZ at 24h intervals, replacing culture medium with vector-containing supernatant at a 1:1 ratio. Similarly treated Jurkat cells with empty vector were used as controls. At 72 h after the second transduction, the cells were subjected to puromycin (5 μg/ml, Sigma-Aldrich) selection. HBZ-positive cells were then subjected to limiting-dilution cloning. HBZ expression was verified by western blot analysis as described below.

### Western Blotting

HBZ proteins were precipitated from both nuclear and cytoplasmic protein fractions using anti-HBZ 4D4-F3 mAb as described above. The precipitated proteins were resolved on 9% SDS-PAGE and analyzed by Western blotting with the anti-HBZ 4D4-F3 mAb, anti-DDX5 rabbit polyclonal antibody (ab21696, ABCAM), and anti-DDX17 rabbit polyclonal antibody (ab24601, ABCAM) followed by HRP-conjugated goat anti-mouse IgG (Invitrogen) (1:500) to detect HBZ and HRP-conjugated goat anti-rabbit IgG (1:1000) to detect DDX5 and DDX17.

### RNA Seq analysis

The RNA-seq analyses were performed with total RNA extracted from ATL-2, Jurkat cells expressing or not HBZ and MOLT-4, in triplicates. Directional (rRNA removal) and mRNA library preparation were generated at Novogene (Hong Kong, China) using Stranded RNA Sample Pre Kit (Illumina) and sequenced by NovaSeq PE150 (15G raw data per sample). Each sample had in average of 6x10^7^ paired-end pairs of reads. RNA-seq data were analyzed using FaRLine, a computational program dedicated to analyzing alternative splicing with FasterDB database (29). Gene expression level in each sample was calculated with HTSeq-count (v0.7.2), and differential expression between conditions was computed with DESeq2 (v1.10.1) (|log2-FoldChange| ≥ 0.4; p ≤0.05) (30, 31).

### RT-PCR

Total RNA was extracted from cell pellets using TRIzol reagent (Thermo Fisher Scientific Waltham, MA). cDNA was synthesized from 1μg total RNA using iScript cDNA Synthesis Kit (Bio-Rad). 1μg of cDNA were amplified by PCR using 2x PCR Bio Taq Mix (PCR Biosystems) according to the manufacturer protocol, and used for subsequent RT-PCR, using the primers listed in **Table 1**. PCR products were analyzed by agarose gel electrophoresis with ethidium bromide under UV light. The RT-PCR experiments were performed in replicates.

**Table 1:** List of the primer sequences used in RT-PCR.

Gene	Exons	Primer sequences
ASXL1	Exon 10 FW	5’ GTG ATG CTG CCT CGA GTT G 3’
	Exon 12 RW	5’ CAC GGA GGT TGG TGT TGA C 3’
MALT1	Exon 6 FW	5’ GTG CCT TAT GTG GAT TTG GAA C 3’
	Exon 9 RW	5’ GGC TGG TCA GTT GTT TGC TC 3’
NKTR	Exon 4 FW	5’ GTT CTA CGT TCC ATC GTG TG 3’
	Exon 7 RW	5’ CCA TTG GTA TGT TTC CCT CG 3’
GUF1	Exon 8 FW	5’ GGA GTC TTG AAT CCT AAT GAG 3’
	Exon 10 RW	5’ GAA CGG TCA CAC TGG AAT C 3’
STX16	Exon 3 FW	5’ GGC CAG CCT TCA TGA CAA G 3’
	Exon 5 RW	5’ CTG GGA TCT TTC CTC TCG ATT C 3’
TSTD1	Exon 1 FW	5’ GTT GCT ACG CGC ACC ATG 3’
	Exon 3 RW	5’ CTG GCT CCA TCT GCA GAG 3’
FNIP1	Exon 6 FW	5’ CAA GAT AGT CTT GAA TTC ATC AAT C 3’
	Exon 8 RW	5’ CTA TGC CAC TGT CTC TGT CCT C 3’
CRAMP1L	Exon 16 FW	5’ CAG GAC TCT ACT GGA ACT C 3’
	Exon18 RW	5’ CGG TAT GGA GAG GCC ATT C 3’

### Immunofluorescence and Confocal Microscopy

ATL-2, Jurkat-HBZ expressing cells and Jurkat cells were cultured on glass coverslips precoated with poly-Lysine (0.3 mg/ml) for 3 hrs. The cells were then fixed with cold methanol 3 min at -20°C and blocked with 1% bovine serum albumin (BSA) in 1× PBS for 1 h at room temperature (RT). To assess co-localization of HBZ with RNA helicases DDX5 and DDX17, cells were then co-stained overnight at 4°C with anti-HBZ 4D4-F3 mAb and either anti-DDX5 rabbit polyclonal antibody (1:1000) (ab21696, ABCAM) or anti-DDX17 rabbit polyclonal antibody (1:250) (ab24601, ABCAM) diluted in 1×PBS buffer containing 0.1% BSA. The slides were then washed three times with 1× PBS and incubated for 2h at RT in the dark with the following secondary antibodies: goat anti-mouse IgG (H+L) coupled to Alexa Fluor 546 (1:400) to detect HBZ and goat anti-rabbit IgG (H+L) conjugated to Alexa Fluor 488 (1:400) to detect DDX5 and DDX17. The nuclei were then stained by incubating the cells with DRAQ5 Fluorescent Probe (1:1000) (Thermo Fisher Scientific) for 30 min at RT in dark.

After washing, the coverslips were mounted on glass slides (Thermo Fisher Scientific) with the Fluor Save reagent (Calbiochem) and examined by a confocal laser scanning microscope (Leica TCS SP5, 63× original magnification, numerical aperture 1.25). Images were acquired and analyzed by LAS AF Lite Image (Leica Microsystems). Region of Interest (ROI) drawn along the mid-nucleus level of the merge images was represented by ROI diagrams. Each ROI diagram corresponds to a single Z-plan.

## Results

### Nuclear HBZ interactome in ATL-2 leukemic cells consists of proteins mostly involved in RNA processing

**Figure 1:**
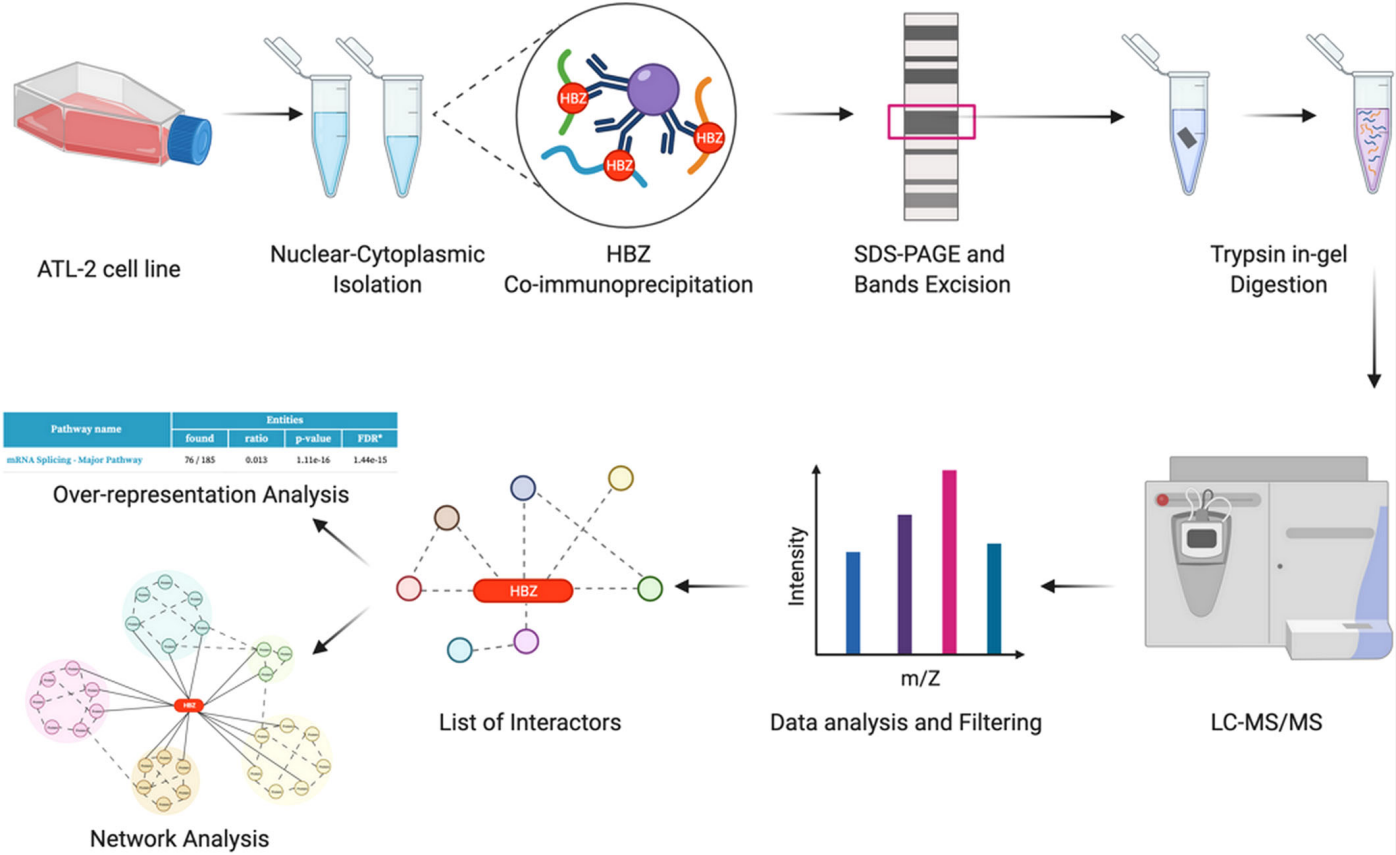
Identification of potential HBZ interactors. Schematic workflow of the proteomic analysis performed in this study (graphics using BioRender.com).

ATL-2 leukemic cells were used as a prototype of exclusive HBZ nuclear localization (19, 22). Endogenous HBZ was immunoprecipitated from the nuclear and cytoplasmic extracts. The immunoprecipitated complexes were run on SDS-PAGE to separate the protein interactors based on their electrophoretic mobility. Gel was stained with colloidal Coomassie (**Supplementary Figure 1**) and bands were cut, trypsin digested, and the obtained peptide mixtures were analyzed by nano-LC-MS/MS for protein identification. The complete procedure is depicted in **Figure 1**.

**Figure 2:**
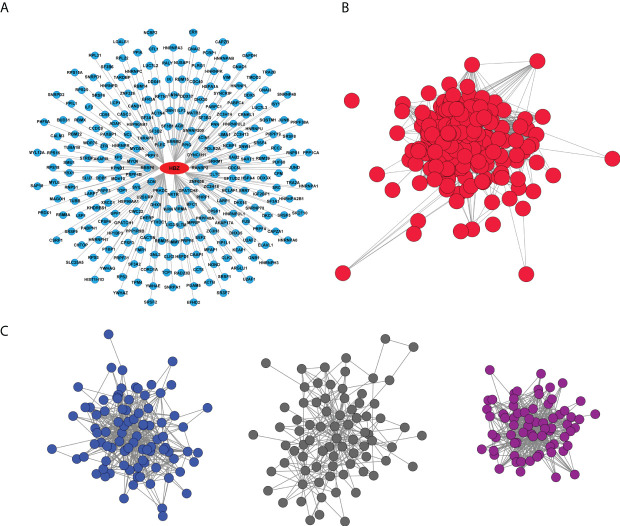
HBZ protein interaction network. **(A)** Graphic representation of endogenous HBZ interacting nuclear factors in the ATL-2 leukemic cells. **(B)** Reciprocal protein-protein interaction network of the 249 HBZ interactors. **(C)** Subnetworks from HBZ interactors network clustered by the GLay algorithm: RNA splicing sub-cluster (dark blue), JAK/STAT signaling pathway sub-cluster (dark grey), non-mediated mRNA decay (NMD) sub-cluster (violet).

Two-hundred forty-nine potential HBZ protein partners were identified in the nucleus of ATL-2 leukemic cells, (**Figure 2A**, **Supplementary Data 1-1**) whereas none were identified in the cytoplasmic fraction as expected by the absence of HBZ in this subcellular compartment. An over-representation analysis of these data by using Reactome as reference database was then performed to get deeper inside into the cellular pathways specifically targeted by nuclear HBZ. Interestingly, among the top 10 significant pathways, the main enriched biological pathway was related to mRNA splicing and processing (**Supplementary Data 1-2**).

We then proceeded to generate the biological network of the 249 identified HBZ interactors by querying the Intact database *via* Cytoscape. Interestingly, the network depicted 24325 direct associations between 7154 proteins (**Supplementary Figure 2**). The experimentally identified HBZ-interacting proteins were then extrapolated from the whole original network (**Figure 2B**). Of relevance, most of the nodes (241 out of 249) resulted to be interconnected (1957 edges) and part of a predominant group.

To reveal pathways that could be hidden in the analysis of the whole network we further clustered it into subnetworks using the GLay algorithm, highlighting three main subclusters (**Figure 2C**), that were subsequently analyzed by an over-representation analysis, to specifically identify their major functions. In the first cluster (dark blue nodes, **Figure 2C**) we identified 88 interacting proteins with 486 associations, all linked to RNA processing. This cluster includes most of the associations and is mainly related to the spliceosome, confirming the over-representation analysis of the 249 HBZ interactors (**Supplementary Data 1-2**). The second cluster (dark grey nodes, **Figure 2C**) identified 78 interacting proteins with 264 associations, related mostly to the JAK-STAT signaling-dependent gene expression or RhoGTPase effectors as indicated by the over-representation analysis (**Supplementary Data 1-3**). The third cluster (violet nodes, **Figure 2C**) identified 68 interactors with 466 interactions mainly involved in RNA processing, particularly Nonsense mediated mRNA decay (NMD) and Processing of Capped Intron-Containing Pre-mRNA, again confirming the major pathways identified by the over-representation analysis (**Supplementary Data 1-4**).

Collectively, the over-representation analysis of the clustering of the nuclear HBZ interactome has demonstrated the tight association of the nuclear HBZ with proteins mainly involved in mRNA splicing and processing.

### HBZ expression is associated with alterations in the host transcriptomic profile

**Figure 3:**
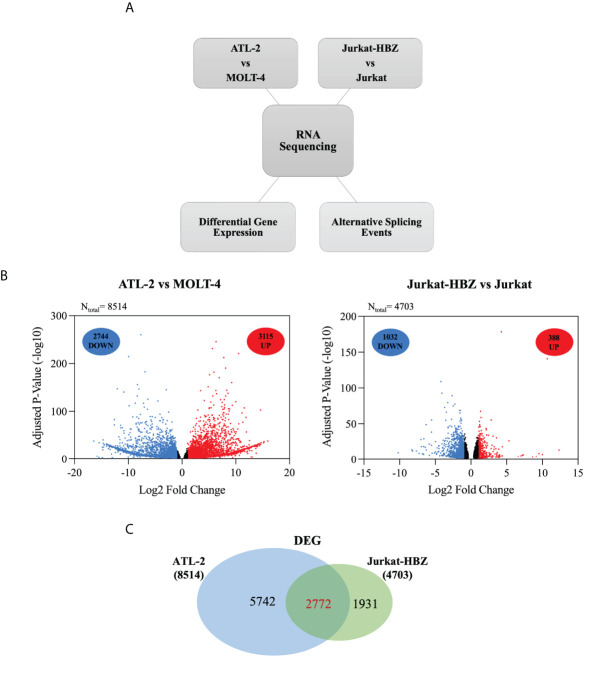
Comparative transcriptome analysis of HTLV-1-derived ATL-2 vs non HTLV-1-derived MOLT-4 leukemic cells, and of Jurkat-HBZ vs Jurkat cells. **(A)** Overview of the strategy used to identify the changes in both the cellular transcriptome and alternative splicing landscape associated with HTLV-1-derived ATL-2 cells or with Jurkat-HBZ transfected cells. **(B)** Volcano plot of significantly up-regulated genes (red dots) and down-regulated genes (blue dots) with |log2fold-change|>1.0 (p < 0.05) in ATL-2 vs MOLT-4 cells (left panel) or Jurkat-HBZ expressing cells vs Jurkat cells (right panel). Dark dots represent the dysregulated genes with 0.4<|log2fold-change|<1.0 (p < 0.05). **(C)** Venn diagram showing differentially expressed genes (DEG) that are shared between ATL-2 and Jurkat-HBZ expressing cells.

To assess whether the HBZ interaction with factors of RNA splicing and stability (cumulatively 63%) could influence the host gene expression profile, we performed high throughput RNA sequencing (RNA-seq) of ATL-2 compared to a non-HTLV-1-derived leukemic T cell, namely MOLT-4, and in parallel RNA-seq of an isogenic cell system composed of a similarly non HTLV-derived leukemic T cell, Jurkat, and its HBZ expressing derivative, Jurkat-HBZ. Interestingly, in contrast to ATL-2 leukemic cells, Jurkat-HBZ cells express HBZ both in the nucleus and in the cytoplasm, mimicking the HBZ-subcellular localization of ATL patients cells (22) (**Supplementary Figure 3**). While ATL-2 versus MOLT-4 could give information on the global effect of HTLV-1 infection leading to leukemia, in which HBZ is a component, the comparison of Jurkat vs Jurkat-HBZ could provide a picture of the exclusive participation of HBZ to the alteration of gene expression in a leukemic background. In the above cellular models, we assessed both differentially expressed genes (DEGs) and alternative splicing events (ASEs) (**Figure 3A**). The common features in the two distinct systems (ATL-2 versus Jurkat-HBZ) could then be taken as a suggestive association of HBZ to the alteration of RNA splicing and stability.

Comparative analysis of ATL-2 cells with uninfected leukemic MOLT-4 cells revealed the existence of 8514 differentially expressed genes (DEGs) (|log2fold-change|>0.4; p<0.05) (**Supplementary Data 2-1**), 5859 of which with a minimum absolute fold change of 2 (|log2fold-change|>1.0; p<0.05). As shown in the volcano plot (**Figure 3B**, left panel), 3115 genes out of 5859 were upregulated (red dots) whereas the other 2744 genes were downregulated (blue dots). The remaining dysregulated genes with an absolute fold change ranging between 0.4 and 1 are represented in dark dots.

Interestingly, comparative analysis of Jurkat-HBZ with Jurkat control cells identified 4703 differentially expressed genes (|log2fold-change>0.4|; p<0.05) (**Supplementary Data 2-2**). Again, by selecting the dysregulated genes with an absolute fold change of 2 (|log2fold-change|>1.0; p<0.05), we found that the expression of HBZ in Jurkat cells (**Figure 3B**, right panel) was associated with the upregulation of 388 genes (red dots) and the downregulation of 1032 genes (blue dots) for a total of 1420 altered genes, consistent with several studies demonstrating that HBZ can act as a transcriptional repressor (32, 33). The remaining dysregulated genes with an absolute fold change ranging between 0.4 and 1 are represented in dark dots. Importantly, GO DAVID analysis on DEGs identified RNA transport and RNA polymerase, as cellular pathways specifically enriched in Jurkat-HBZ cells providing a further potential link of HBZ to alteration of the RNA metabolism (p<0.001; FDR<0.001) (**Supplementary Data 2-3**).

By comparing the transcriptional profile of ATL-2 and Jurkat-HBZ cells, we observed 2772 commonly differentially expressed genes (**Figure 3C**, **Supplementary Data 2-4**) indicating that 59% of HBZ-dependent transcriptionally regulated genes are also differentially expressed in ATL-2 cell line.

**Figure 4:**
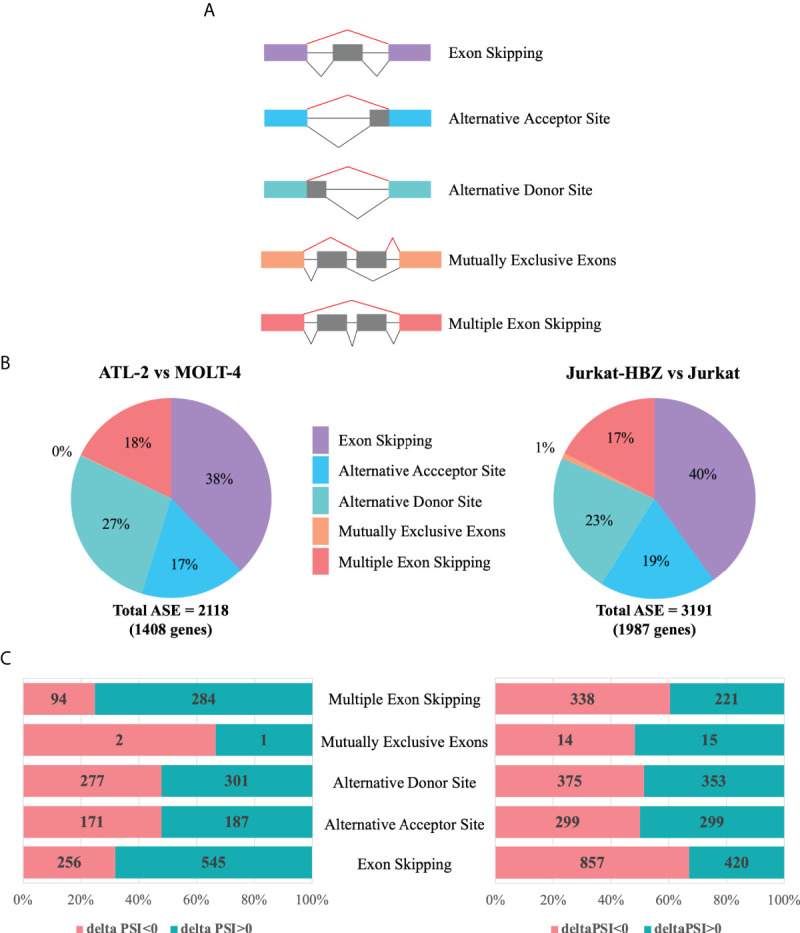
Alternative splicing modifications upon HTLV-1-derived leukemic transformation or HBZ expression. **(A)** Schematic representation of five main alternative splicing events (lines in red), listed in the right. **(B)** Alternative Splicing Events (ASEs) detected in ATL-2 (left) and Jurkat-HBZ (right) cells. **(C)** Exclusion (deltaPSI<0) and Inclusion (deltaPSI>0) number of ASEs witnessed in ATL-2 (left) and Jurkat-HBZ (right) cells are indicated within the columns of the bar graph.

We then investigated whether differentially expressed genes in ATL-2 and Jurkat-HBZ cells were also target of alternative splicing events. Firstly, we evaluated the total impact of HBZ expression on host cellular splicing machinery by statistically computing splicing events using FARLine pipeline. We mainly focused on 5 alternative splicing events: exon skipping, alternative acceptor site, alternative donor site, mutually exclusive exons, and multiple exons skipping (**Figure 4A**), which we quantified as Percent Spliced In (PSI) considering positive the splicing events with |deltaPSI|>0.1 and p<0.05. Our analysis identified 2118 and 3191 ASE corresponding to 1408 and 1987 genes in ATL-2 (**Figure 4B**, left panel) and Jurkat-HBZ cells (**Figure 4B**, right panel), respectively (**Supplementary Data 3**). In both cells most of the alternative splicing events correspond to exon skipping, either single (38% in ATL-2 and 40% in Jurkat-HBZ) or multiple exon skipping (18% in ATL-2 and 17% in Jurkat-HBZ). In ATL-2 cell line, inclusion of exons (68%) is the predominant exon skipping event (**Figure 4C**-left panel) as opposed to exclusion of exons (67%) in Jurkat-HBZ cells (**Figure 4C**-right panel). Interestingly, splicing alterations due to alternative donor or receptor site were very similar in percentage between ATL-2 and Jurkat-HBZ. Furthermore, GO-DAVID analysis indicated that HBZ-induced alternatively spliced genes were mainly significantly enriched for T cell receptor signaling pathway and mRNA surveillance pathway (p<0.001) (**Supplementary Data 3-13**). To gain insight into the biological outcomes of the splicing alterations, we carried out exon-ontology analysis that estimate enrichment in protein features encoded by exons regulated by HBZ (34). The **Supplementary Data 3-14** shows that these exons coded for regions involved in functionall validated binding domains of protein and nucleic acid. Overall, these findings demonstrated that HTLV-1 leukemic cells are characterized by significative perturbation of exon content of multiple transcripts, characteristic that is also found upon HBZ expression.

**Figure 5:**
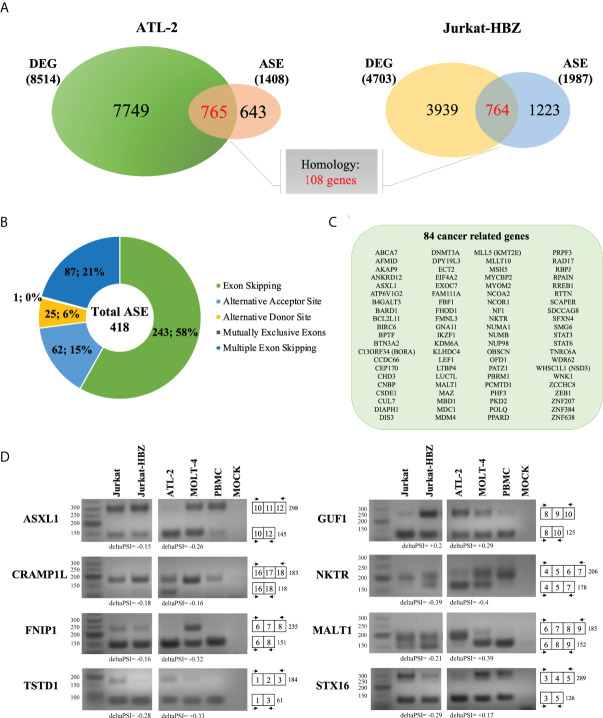
Comparative analysis between ATL-2 and Jurkat-HBZ cells. **(A)** Venn diagrams showing differentially expressed and alternatively spliced genes that are shared between ATL- 2 (left) and Jurkat-HBZ (right) cells. **(B)** Donut pie representing the number of genes undergoing ASEs shared between ATL-2 and Jurkat-HBZ cells. **(C)** List of shared cancer related genes alternatively spliced in ATL-2 and Jurkat-HBZ cells. **(D)** RT-PCR validation of microarray-predicted exon events in ATL-2 as compared to MOLT-4 and healthy PBMCs, and in Jurkat-HBZ cells compared to Jurkat cells. RNA seq derived delta PSI values are indicated below each PCR reaction. Gene designation is on the left and amplification boundaries are on the right with numbers representing the specific exons. The arrows represent the position of the designed primers.

Subsequently, we assessed the existence of a possible correlation between alternative splicing and gene expression. In fact, 765 and 764 genes were identified to be alternatively spliced and differentially expressed in ATL-2 (**Figure 5A**, left panel) and Jurkat-HBZ cells (**Figure 5A**, right panel), respectively (**Supplementary Data 4-1,2**). Thus, about half of the genes that undergo alternative splicing are also differentially expressed in ATL-2 cells (54%; 765/1408). A consistent number of transcripts harboring ASE were also differentially expressed in Jurkat-HBZ cells (38%; 764/1987). By comparing ATL-2 and Jurkat-HBZ cells, 108 genes were still in common between the two cells at the splicing and expression level. Although not quantitatively altered, some protein-encoding transcripts were structurally altered, uncovering a new, hitherto unexplored, layer of regulation.

By comparing the transcriptional profile of ATL-2 and Jurkat-HBZ cells, we found 418 alternative splicing events in common involving 403 genes in both ATL-2 and Jurkat-HBZ cells. Most of these alternative splicing events corresponded to exon skipping process (58%) (**Figure 5B**, **Supplementary Data 4-4**). Further highlighting the impact of HBZ in the leukemic process, we observed that 84 out of the 403 shared could be identified as cancer genes (**Figure 5C**) (ncg.kcl.ac.uk). (**Supplementary Data 4-5**) Notably, only 27% (23/84) and 3.5% (3/84) cancer related genes were differentially expressed in ATL-2 and Jurkat-HBZ cells, respectively (|log2FC|>1, p<0.05, **Supplementary Figure 4**), indicating that the majority of these HBZ splicing targets are not affected in their expression level. Furthermore, changes in gene expression levels in ATL-2 were not related to HBZ expression as demonstrated in Jurkat-HBZ cells (**Supplementary Figure 4**), suggesting that HBZ-mediated splicing effects affect transcripts independently of their expression levels. Interestingly, among the identified cancer genes, MALT1 has been recently demonstrated to be involved in the regulation of NF-κB signaling in HTLV-1 mediated leukemogenesis, representing a promising therapeutic target gene for ATL (35).

We then validated the RNA-seq analysis by RT-PCR selecting 8 genes commonly spliced in ATL-2 and Jurkat-HBZ expressing cells. Of those, gene expression levels of STX16 and TSTD1 reached the significant threshold of gene deregulation in HBZ positive Jurkat cells but not in ATL2. The other genes were not affected at the gene expression level in both ATL2 and Jurkat cells expressing HBZ (|log2FC|>1, p<0.05, **Supplementary Figure 4**). Specific primers were designed to verify the predicted ASEs and MOLT-4 and Jurkat T cells were used as internal controls for ATL-2 and Jurkat-HBZ cells, respectively. Consistent with the findings predicted by RNA-seq analysis in ATL-2, we observed exon exclusions in 5 transcripts (ASXL1, NKTR, STX16, FNIP1, CRAMP1L) whereas exon inclusion was detected for MALT1 and GUF1 transcripts (**Figure 5D**). In Jurkat-HBZ cells, exon exclusion was verified in 5 tested genes (MALT1, NKTR, STX16, TSTD1, FNIP1) while exon inclusion was shown in one tested transcript (GUF1) (**Figure 5D**). The anomalous pattern of the ASEs in ATL-2 and Jurkat-HBZ cells was further highlighted by comparison with normal PBMC where, as expected, we observed the predominance of single transcript for the reference gene analyzed. Taken together our analysis shows that although some differences of the host splicing landscape were observed in ATL-2 and Jurkat-HBZ expressing cells, the two cells share common target exons that are similarly affected, suggesting that HBZ specifically induces these qualitative alterations in gene expression.

### HBZ shares target exons with various host splicing factors

**Figure 6:**
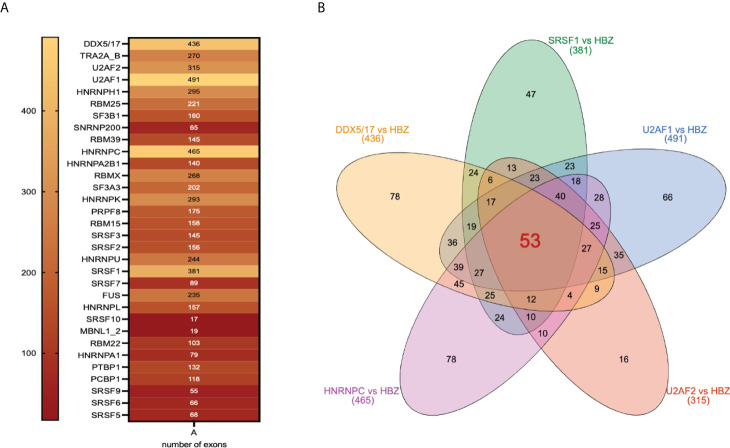
Shared target exons between HBZ and HBZ-interacting splicing factors. **(A)** 32 different splicing factors interacting with HBZ are shown with inside the numbers of target exons they share with HBZ target exons. **(B)** Venn diagram showing the number of inter-common target exons between HBZ and each of the major splicing factors: DDX5/17, SRSF1, U2AF1, U2AF2, and HNRNPC.

On the basis of the results presented above, it was important to assess also whether HBZ and its interacting splicing factors identified by proteomics could target common exons. We then performed a comparative analysis between HBZ-regulated alternative splicing events and the target exons of 32 HBZ-interacting splicing factors (36) (**Figure 6A**). Interestingly, we found that 53 exons targeted by HBZ, were commonly spliced by the most important splicing factors, namely DDX5, DDX17, U2AF1, U2AF2, SRSF1, and HNRNPC, (**Figure 6B**, **Supplementary Data 5**), supporting the role of HBZ in the alteration of the alternative splicing of host transcripts. It remains to be established whether HBZ directly affects exons splicing or acts by activating the interacting splicing factors.

### HBZ partially co-localizes and interacts with DDX5 and DDX17 in ATL-2 leukemic cells and Jurkat-HBZ expressing cells

To further substantiate the interaction of HBZ with crucial splicing factors and extend the information both on the extent and the subcellular compartments of the interaction, we performed additional biochemical and confocal microscopy analyses focusing on RNA helicases DDX5 and its paralog DDX17, which have been recently reported to be involved in alternative splicing on cellular transcripts after the NF-κB activation by HTLV-1 Tax-1 (37).

**Figure 7:**
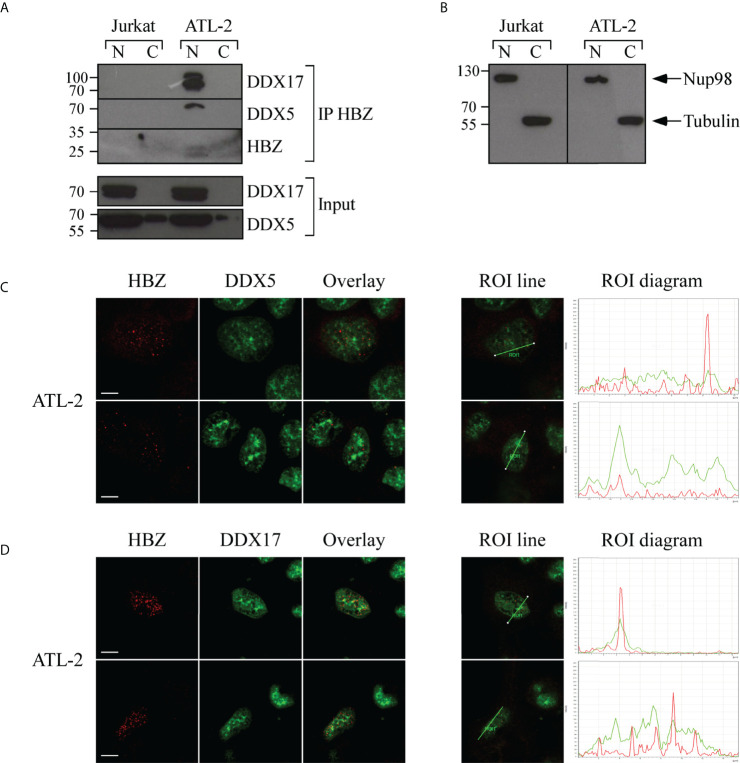
*In vivo* interaction and colocalization of endogenous HBZ with DDX5 and DDX17 in ATL-2 cells. **(A)** HBZ interaction with DDX5 and DDX17 in ATL-2 cells was assessed by co-immunoprecipitation assay. Nuclear and cytoplasmic protein extracts of Jurkat and ATL-2 cells were immunoprecipitated with the anti-HBZ 4D4-F3 mAb (IP HBZ) and the eluted material analyzed by western blotting for the presence of co-immunoprecipitated DDX5 and DDX17 molecules using specific monoclonal antibodies. Input levels of respective DDX5 and DDX17 are represented in the lower panels. **(B)** The purity of nuclear and cytoplasmic fraction extracted fractions was verified by western blot (input) using ten percent of the nuclear and cytoplasmic protein extracts and antibodies specific for the nuclear protein Nup98 or β-tubulin for cytoplasmic protein. N, nuclear; C, cytoplasmic. ATL-2 cells were reacted in a pairwise combination with the 4D4-F3 anti-HBZ mAb and either polyclonal rabbit anti-DDX5 **(C)** or anti-DDX17 **(D)** antibodies. Anti-HBZ mAb was revealed by Alexa fluor 546-labeled goat anti-mouse IgG (red), whereas the other rabbit antibodies were revealed by Alexa fluor 488-labeled goat anti-rabbit antibodies (green). The colocalization is represented in the overlay panels (yellow). The region of interest (ROI) drawn along mid-nucleus level of the merge image is represented by red (HBZ) and green (RNA helicases) peaks in the histogram. At least 200 cells were analyzed. All scale bars are 5 µm.

In addition, in Jurkat-HBZ expressing cells we have found that among the HBZ-induced alternatively spliced exons, 436 are also target exons of DDX5 and DDX17. Thus, we first re-assessed the association between endogenous HBZ and DDX5 or DDX17 in ATL-2 cells. Cell extracts were first incubated with the 4D4-F3 anti-HBZ mAb and the resulting immunoprecipitate run on SDS-PAGE. Gels were blotted onto membranes which were then incubated with antibodies specific for either DDX5 or DDX17. As shown in **Figure 7A**, both DDX5 and DDX17 were clearly found in the anti-HBZ immunoprecipitate of the ATL-2 nuclear fraction. The relatively low detection of HBZ in western blots after immunoprecipitation reflected both the low amount of endogenous HBZ in ATL-2 cells and a partial loss of HBZ-specific epitopes detectable with the 4D4-F3 antibody in denaturing conditions. The purity of the nuclear-cytoplasmic extraction is represented in (**Figure 7B**). Subsequently, we performed an extensive confocal microscopy analysis to assess the subcellular distribution of these proteins and to verify the extent of a possible colocalization with HBZ. As previously shown, in the ATL-2 leukemic cells HBZ was localized exclusively in the nucleus with a dot-like shape (**Supplementary Figure 3B**). Similarly, DDX5 (**Figure 7C**) and DDX17 (**Figure 7D**) were also localized in the nucleus but with larger dots and more diffuse distribution. Interestingly, colocalization between HBZ and either DDX5 or DDX17 was partial and observed in discrete nuclear areas as assessed by region of interest (ROI) scanning (**Figure 7C** and **Figure 7D**, ROI lines, and ROI diagram columns).

**Figure 8:**
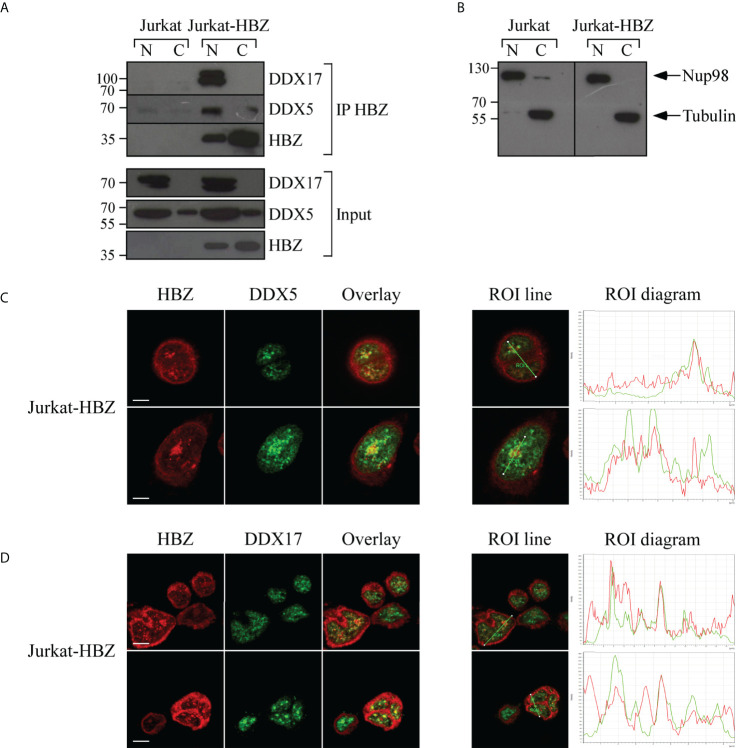
*In vivo* interaction and colocalization of exogenous HBZ with DDX5 and DDX17 in Jurkat-HBZ cells. **(A)** HBZ interaction with DDX5 and DDX17 in Jurkat-HBZ cells was assessed by co-immunoprecipitation assay. Nuclear and cytoplasmic protein extracts of Jurkat and Jurkat-HBZ cells were immunoprecipitated with anti-HBZ 4D4-F3 mAb (IP HBZ) and the eluted material was analyzed by western blotting for the presence of co-immunoprecipitated DDX5 and DDX17 molecules using specific monoclonal antibodies. Input levels of respective HBZ, DDX5 and DDX17 are represented in the lower panels. **(B)** The purity of nuclear and cytoplasmic extracted fractions was verified by western blot (input) using ten percent of the nuclear and cytoplasmic protein extracts and antibodies specific for the nuclear protein Nup98 or β-tubulin for cytoplasmic protein. N, nuclear; C, cytoplasmic. Jurkat-HBZ expressing cells were reacted in a pairwise combination with the 4D4-F3 anti HBZ mAb antibody, and either polyclonal rabbit anti-DDX5 **(C)** or anti-DDX17 **(D)** antibodies. Anti-HBZ mAb was revealed by Alexa fluor 546-labeled goat anti-mouse IgG (red), whereas the other rabbit antibodies were revealed by Alexa fluor 488-labeled goat anti-rabbit antibodies (green). The colocalization is represented in the overlay panels (yellow). The region of interest (ROI) drawn along mid-nucleus level of the merge image is represented by red (HBZ) and green (RNA helicases) peaks in the histogram. At least 300 cells were analyzed. All scale bars are 5 µm.

The association between HBZ and DDX5 and DDX17 was also evident in Jurkat-HBZ cells. As shown in **Figure 8A**, both DDX5 and DDX17 were coimmunoprecipitated with HBZ thus confirming the results obtained with ATL-2 leukemic cell line. It is important to note a lower but distinct interaction of HBZ and DDX5 in the cytoplasmic fraction of Jurkat-HBZ cells. As stated above, these cells express HBZ not only in the nucleus but also in the cytoplasm (**Supplementary Figure 3B**). Thus the cytoplasmic interaction of DDX5 with HBZ confirms the existence of the dual sub-cellular localization of the RNA helicase as previously shown (38). The purity of the nuclear-cytoplasmic extraction is represented in (**Figure 8B**). We then assessed by confocal microscopy the extent of the colocalization. As previously found in ATL-2 cells, nuclear HBZ partially colocalized with both DDX5 (**Figure 8C**) and DDX17 (**Figure 8D**). The amount of HBZ colocalizing with the two RNA helicases was higher compared to ATL-2 cells (ROI line and ROI diagram columns, **Figures 7C, D**) and this correlated with the higher amount of HBZ expressed in the Jurkat-HBZ cells compared to the ATL-2 cell line.

## Discussion

Previous studies of our group indicated that progression of HTLV-1 infection from asymptomatic carriers to leukemic patients is associated to a unidirectional cytoplasmic-to-nuclear transition of HBZ (19–22). We reasoned that this association might be not only a marker of the leukemic state but also a possible causative event in the process of HTLV-1-mediated oncogenesis. Within this frame, the nuclear translocation of HBZ could be involved in the complex dysregulation of gene expression that is a hallmark of the oncogenic process. A large series of previous investigations had indeed shown that HBZ could bind to various host cell molecules and alter their function, particularly at the level of regulation of gene expression (17). However, a complete and structured work aimed at assessing the whole landscape of endogenous HBZ interaction with host molecules, the HBZ interactome, was still lacking. Although some attempts in this direction have been recently reported (39), the results presented here are the first, to our knowledge, that shed light on the real *in vivo* endogenous HBZ interactome in leukemic cells and specifically on the nuclear HBZ interactome in ATL. From the unprecedented complexity of these interactions, it begins to appear a crucial path of “HBZ partnership” with the mechanism of RNA splicing and RNA stability that are crucial components of the biology of gene expression.

It is known that most oncogenic viruses, including HTLV-1, exploit the host RNA splicing machinery not only to efficiently export and stabilize viral RNA and to produce spliced RNA isoforms for the generation of the viral proteins, but also to manifest their oncogenic potential. Perturbation of host cellular gene expression by altering the splicing landscape represents indeed one crucial aspect of virus-induced carcinogenesis (40) (41). Splicing dysregulation was observed in HTLV-1 infected cells *in vivo*, including non-transformed and ATL cells (42) sharing similar splicing modifications, thereby suggesting that alternative splicing contributes to neoplastic transformation and ATL progression. Tax-1 has been involved in alternative splicing modifications (37, 39). Tax-induced NF-κB promotes significant RelA enrichment in gene bodies that recruits the splicing factor DDX17, which regulates alternative splicing of target exons thanks to its RNA helicase activity (37). More recently, a yeast two-hybrid-based study has suggested that both Tax-1 and HBZ may interfere with various RNA splicing factors and affect the host cell transcriptome (39). However, this approach did not take into account the real endogenous interactions taking place within the infected cell, particularly the leukemic cells. By isolating the endogenous HBZ interactome by means of the first described monoclonal antibody against HBZ, we identified novel HBZ interacting partners, and we generated for the first time a protein network map of HBZ in the nucleus of the ATL-2 leukemic cells. We identified 249 cellular factors specifically associated to the viral oncogene and most of them are involved in RNA processing, mainly in RNA splicing pathway and non-sense mediated RNA decay, suggesting that HBZ may exert its oncogenic properties at least in part by perturbing RNA maturation/processing besides its role already described in the host gene expression machinery.

In following with this idea, we first assessed the gene expression profiles of ATL-2 cells used for MS/MS analysis of HBZ interacting proteins. In order to uncouple the impact of HBZ on host gene expression from similar effect of other viral factors, we used an isogenic cell system composed by the Jurkat T cell line and its HBZ stably expressing derivative Jurkat-HBZ. Overall, 8514 and 4703 genes were found differentially expressed in ATL-2 and Jurkat cells expressing HBZ, respectively. Interestingly, most of differentially expressed genes (DEG) were downregulated, upon HBZ expression in Jurkat cells, in agreement with the evidence that HBZ acts predominantly as a transcriptional repressor (32, 33). Moreover, Gene Ontology analysis of HBZ-dependent DEG in Jurkat-HBZ identified the terms “RNA transport” and “RNA polymerase” two pathways that have critical role in gene expression and RNA processing and maturation, again strongly suggesting that HBZ may contribute to HTLV-1-mediated oncogenesis by significantly impacting on host RNA diversity.

Besides differentially expressed genes, we identified 1408 and 1987 alternative splicing events (ASE) in ATL-2 and Jurkat-HBZ respectively, 418 ASE being shared by both cell lines expressing HBZ. Of note, as previously identified for Tax-1 (37), the majority of HBZ-induced ASE (61%) concerned genes with no significant changes in gene expression level, indicating that HBZ acts on splicing regulation independently of its transcriptional effects. This was particularly noticeable for ASE involved in a subset of 84 genes defined as cancer genes in the Network of Cancer Genes (ncg.kcl.ac.uk), of which only 4 (4.7%) were affected in their whole gene expression level. Interestingly, one of these genes, namely MALT-1, is a crucial regulator of NF-κB pathway in MALT lymphoma and appears to be involved in the regulation of NF-κB activation in HTLV-1 mediated leukemogenesis (35). Here we found that exon 7 of MALT-1 which supports optimal T-cell signalling and activation (43) was differently spliced in both ATL-2 and Jurkat-HBZ cells. In addition, the exon 6 of Natural Killer T-cell Receptor (NKTR), another crucial cancer gene, was excluded in both cellular systems expressing HBZ. The individual, as well as collective, functional impacts of HBZ-induced alternative splicing modifications on persistence and immortalisation of HTLV-1 infected cells will require further elucidation.

Finally, we showed that 53 HBZ-dependent ASE involve exons co-regulated by 6 HBZ-interacting factors identified as critical spliceosome components: DDX5, DDX17, U2AF1, U2AF2, SRSF1, and HNRNPC. Future investigation will address the question of whether HBZ acts directly as a splicing factor on the above shared exons or indirectly *via* activation of the splicing activity of the interacting factor. The *in vivo* interaction of HBZ with crucial splicing factors was further substantiated and extended by biochemical and confocal microscopy analyses of DDX5 and DDX17 which were clearly shown to physically interact with HBZ within the cell and to partially colocalize in the nucleus of both ATL-2 and Jurkat-HBZ cells. Taken together, the above results pinpoint HBZ as a crucial actor in the complex series of events leading to the alteration of splicing involved in ATL. Given the broad functional involvement of RNA helicases in transcription, splicing, nuclear export, and RNA translation (44), future studies should evaluate the combined effects of HBZ interactions on multi-step processes of regulation of gene expression.

Besides its role in proteome diversity, alternative splicing can also modulate gene activity through the production of transcripts containing premature termination codon (PTC), which constitutes potential targets of NMD. One fourth of alternative splicing isoforms shared between human and mouse cells has been shown to contain conserved PTC, suggesting an important functional role in coupling splicing to NMD (45). In tumor cells, including hematologic malignancies, coupling between splicing and NMD has been involved in cell survival (46). Considering the mechanistic relationship between splicing and NMD, together with the role of some HBZ-interacting factors in both regulatory mechanisms, future studies would have to address whether HBZ affects the stability of some mRNA isoforms, and if so, how this influences ATL development.

As mentioned above, we have demonstrated that HBZ is exclusively localized in the cytoplasm of cells of asymptomatic carriers and HAM/TSP patients whereas is frequently found both in the cytoplasm and the nucleus of fresh ATL leukemic cells (22). Interestingly, the HBZ transduced Jurkat cells expresses HBZ both in the cytoplasm and the nucleus (see **Supplementary Figure 2**), thus mimicking the HBZ subcellular distribution frequently observed in ATL patients (22). Jurkat-HBZ cells may thus represent a useful model to assess the role of HBZ in the progression of ATL. Future studies will be focused on the analysis of HBZ interactome in Jurkat-HBZ cells, as well as in additional ATL cell lines more closely mimicking the leukemic process (47) and in fresh leukemic cells (14, 48) to further validate and possibly expand and compare proteomic data of HBZ interactors in other HTLV-1-derived ATL and in cells that are not derived from HTLV-1-induced leukaemia. Moreover, the analysis of the HBZ interactome in the cytoplasm of Jurkat-HBZ may unveil previously undetected HBZ-interacting partners which may explain the molecular correlates of cytoplasmic retention of the viral protein during the transition from non-malignant to malignant phenotype. This in turn may open the way to find possible drug targetable molecules that mediate this transition and add urgently needed new armamentarium to combat this still untreatable deadly disease.

## Data availability statement

The datasets presented in this study can be found in online repositories. The names of the repository/repositories and accession number(s) can be found below:

NCBI Gene Expression Omnibus, GEO accession number GSE206085.

## Author contributions

Conceptualization: GF, RSA. Data curation: GF, MS, TA, MF, MM, II, JL, FM. Formal analysis: GF, MS, TA, MF, MM, II, JL, FM. Funding acquisition: GF, RSA. Investigation: GF, MS, II, JL. Supervision: GF, RSA. Writing – original draft: GF, MS, RSA. Writing – review & editing: GF, MS, TA, MF, MM, II, JL, FM, RSA. All authors contributed to the article and approved the submitted version.

## Funding

Associazione Italiana per la Ricerca sul Cancro AIRC, IG 26195 to GF. European Grant: HepaVAC FP7- Health 2013 to RSA. Ligue Nationale Contre le Cancer to JL. INSERM and the Fondation ARC (PJA20191209513) to FM.

## Conflict of interest

The authors declare that the research was conducted in the absence of any commercial or financial relationships that could be construed as a potential conflict of interest.

## Publisher’s note

All claims expressed in this article are solely those of the authors and do not necessarily represent those of their affiliated organizations, or those of the publisher, the editors and the reviewers. Any product that may be evaluated in this article, or claim that may be made by its manufacturer, is not guaranteed or endorsed by the publisher.

## Supplementary material

Supplementary Figure 1 SDS-PAGE gel stained with Coomassie blue. HBZ immunoprecipitated nuclear extracts obtained from ATL-2 cells (40x10^6^ cells) was loaded on SDS-PAGE (lane 2) after preclearing with protein A-Sepharose/protein G-Agarose beads (lane 3). Lane 1 represents the protein ladder. Gel slices were excised from the gel and processed for mass spectrometry as described in Materials and Methods.

Supplementary Figure 2 HBZ whole interactome network from Intact database. Cytoscape whole network of the interactome of the 249 putative HBZ interactors (red nodes) and their first interactors (light blue nodes).

Supplementary Figure 3 Subcellular localization of HBZ in Jurkat-HBZ expressing cells and in ATL-2. Jurkat-HBZ expressing cells **(A)** and ATL-2 cells **(B)** were stained with the 4D4-F3 anti-HBZ mAb followed by Alexa Fluor 546-conjugated goat anti-mouse IgG antibody (red) and analyzed by confocal microscopy. Specific staining of nucleus compartment was performed by using DRAQ5 fluorescence probe. DIC represents the differential interference contrast image. At least 300 cells were analyzed. All scale bars are 5mm.

Supplementary Figure 4 Differential gene expression of shared alternatively spliced genes in ATL-2 and Jurkat-HBZ. Histograms representing the distribution of log2FoldChange of 84 alternatively spliced cancer related genes in ATL-2 vs MOLT-4 **(A)** and in Jurkat-HBZ vs Jurkat **(B)**.

Supplementary Data Sheet 1 (1) List of HBZ potential protein partners. (2) Over-represented top 10 signaling pathways in 249 putative HBZ interactors. (3) Top 10 enriched pathways for cluster 2 proteins. (4) Top 10 enriched pathways for cluster 3 proteins.

Supplementary Data Sheet 2 (1) Differentially expressed genes in ATL-2 vs MOLT-4. (2) Differentially expressed genes in Jurkat-HBZ cells vs Jurkat cells. (3) GO analysis of HBZ induced DEG. (4) 2772 Common differentially expressed genes between ATL-2 and HBZ expressing Jurkat cells.

Supplementary Data Sheet 3 (1) Total ASE in ATL-2 vs MOLT-4 (2) Exon skipping events in ATL-2 vs MOLT-4 (3) Alternative acceptor site in ATL-2 vs MOLT-4 (4) Alternative donor site in ATL-2 vs MOLT-4 (5) Mutually exclusive exons in ATL-2 vs MOLT-4 (6) Multiple exons skipping in ATL-2 vs MOLT-4 (7) Total ASE in Jurkat-HBZ cells vs Jurkat cells (8) Exon skipping events in Jurkat-HBZ cells vs Jurkat cells (9) Alternative acceptor site in Jurkat-HBZ expressing cells vs Jurkat cells (10) Alternative donor site in Jurkat-HBZ cells vs Jurkat cells (11) Mutually exclusive exons in Jurkat-HBZ expressing cells vs Jurkat cells (12) Multiple exons skipping in Jurkat-HBZ cells vs Jurkat cells (13) GO-DAVID analysis of HBZ-induced ASEs (14) Exon ontology analysis of HBZ-induced ASEs

Supplementary Data Sheet 4 Common Genes that are differentially expressed and alternatively spliced in ATL-2 (1) and in Jurkat-HBZ cells (2). (3) Common alternative splicing genes between ATL-2 and Jurkat-HBZ cells. (4) Cancer genes that are splicing targets shared between ATL-2 and Jurkat-HBZ expressing cells.

Supplementary Data Sheet 5 List of target exons shared between HBZ and 32 different splicing factors interacting with HBZ.
