# Supplementary figures and images for: The endogenous HBZ interactome in ATL leukemic cells reveals an unprecedented complexity of host interacting partners involved in RNA splicing
Source: Front Immunol. 2022 Aug 1;13:939863. doi: 10.3389/fimmu.2022.939863 (PMC9376625; doi:10.3389/fimmu.2022.939863)

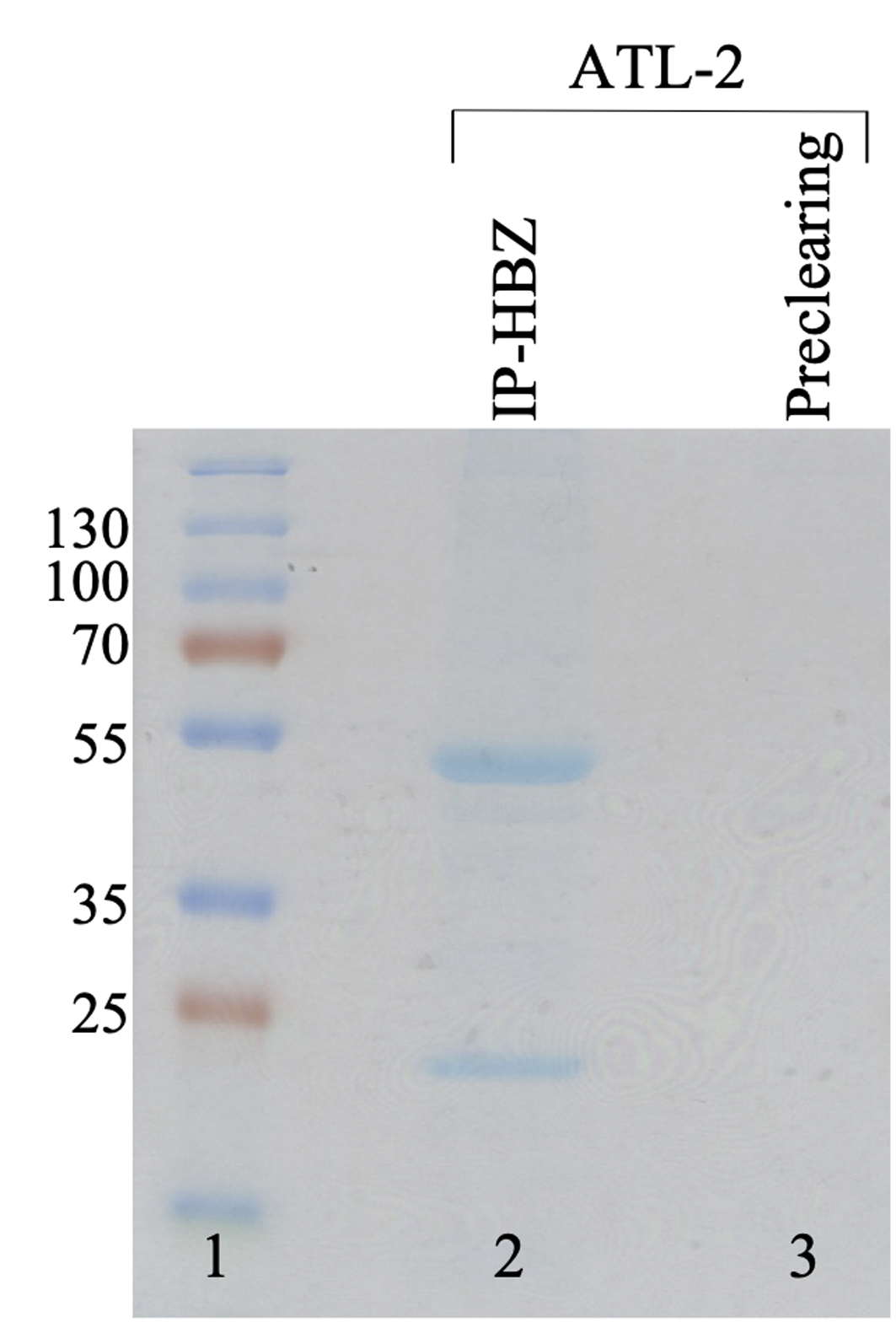

Supplement: Supplementary Figure 1 — SDS-PAGE gel stained with Coomassie blue. HBZ immunoprecipitated nuclear extracts obtained from ATL-2 cells (40x106 cells) was loaded on SDS-PAGE (lane 2) after preclearing with protein A-Sepharose/protein G-Agarose beads (lane 3). Lane 1 represents the protein ladder. Gel slices were excised from the gel and processed for mass spectrometry as described in Materials and Methods. [file Image_1.tif]

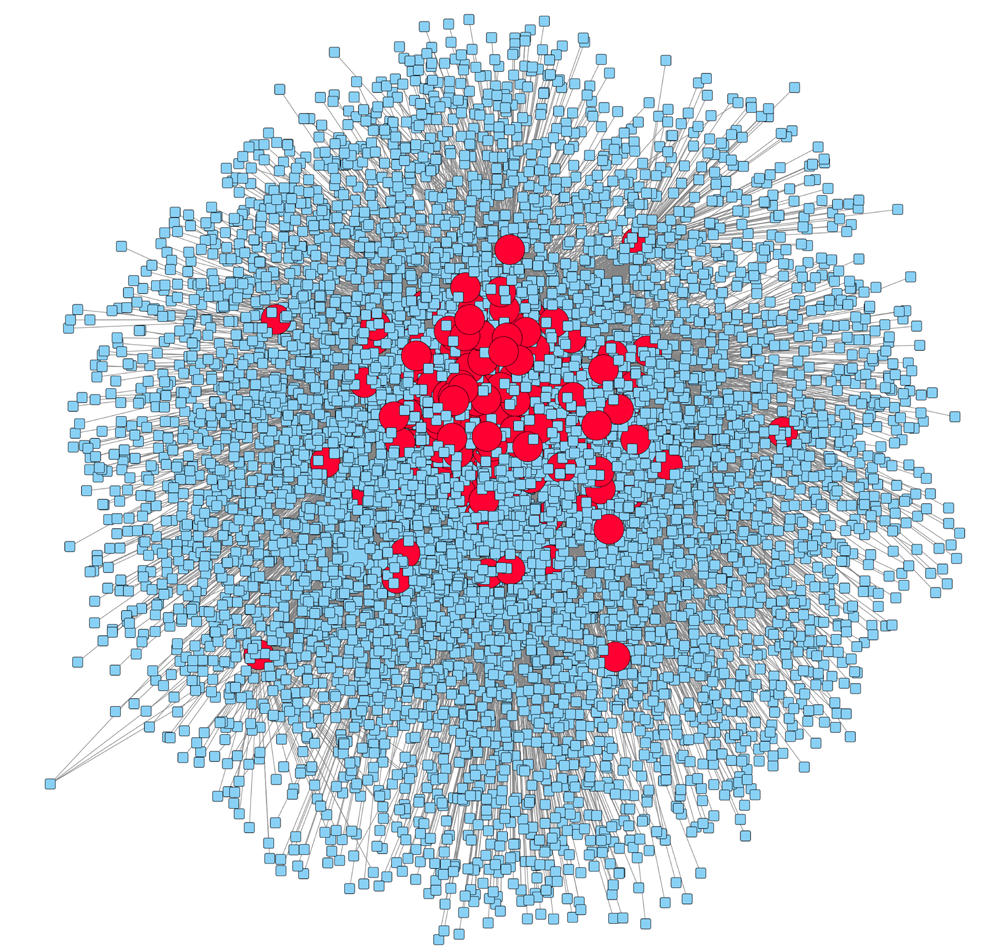

Supplement: Supplementary Figure 2 — HBZ whole interactome network from Intact database. Cytoscape whole network of the interactome of the 249 putative HBZ interactors (red nodes) and their first interactors (light blue nodes). [file Image_2.tif]

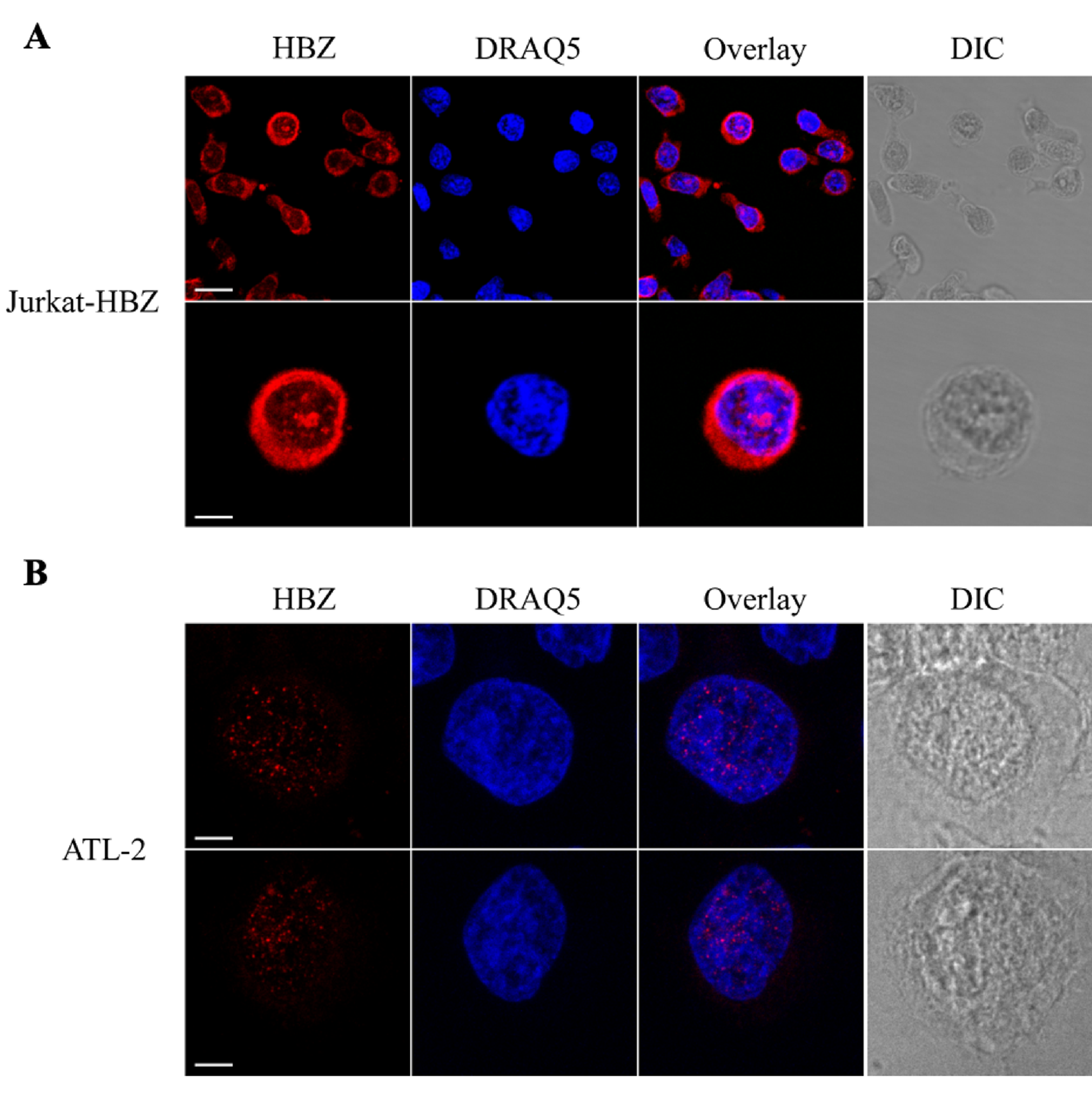

Supplement: Supplementary Figure 3 — Subcellular localization of HBZ in Jurkat-HBZ expressing cells and in ATL-2. Jurkat-HBZ expressing cells (A) and ATL-2 cells (B) were stained with the 4D4-F3 anti-HBZ mAb followed by Alexa Fluor 546-conjugated goat anti-mouse IgG antibody (red) and analyzed by confocal microscopy. Specific staining of nucleus compartment was performed by using DRAQ5 fluorescence probe. DIC represents the differential interference contrast image. At least 300 cells were analyzed. All scale bars are 5mm. [file Image_3.tif]

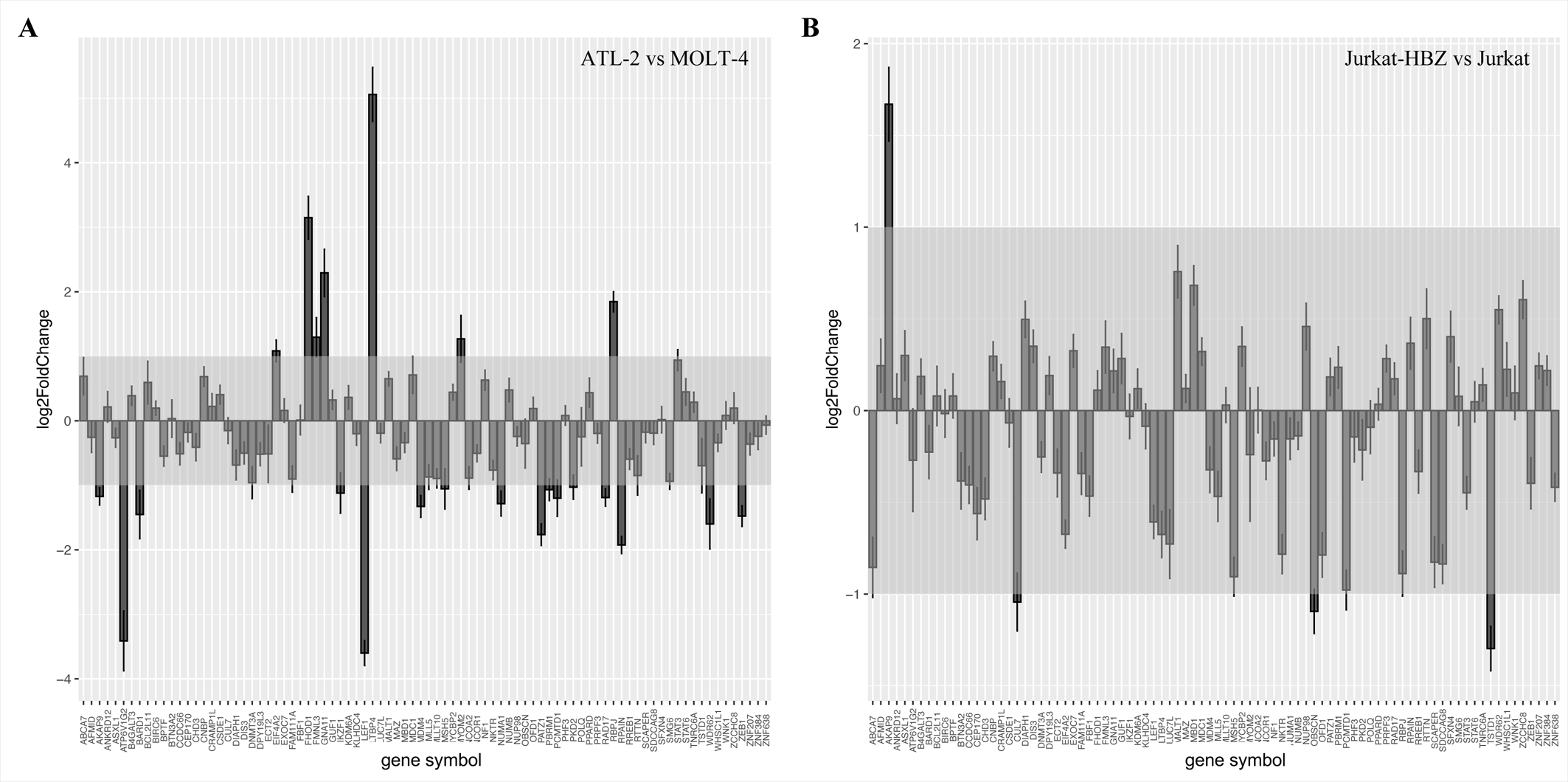

Supplement: Supplementary Figure 4 — Differential gene expression of shared alternatively spliced genes in ATL-2 and Jurkat-HBZ. Histograms representing the distribution of log2FoldChange of 84 alternatively spliced cancer related genes in ATL-2 vs MOLT-4 (A) and in Jurkat-HBZ vs Jurkat (B). [file Image_4.tif]
